# Hepatitis E Virus in Livestock—Update on Its Epidemiology and Risk of Infection to Humans

**DOI:** 10.3390/ani13203239

**Published:** 2023-10-17

**Authors:** Hanna Turlewicz-Podbielska, Agata Augustyniak, Jarosław Wojciechowski, Małgorzata Pomorska-Mól

**Affiliations:** 1 Department of Preclinical Sciences and Infectious Diseases, Poznan University of Life Sciences, Wolynska 35, 60-637 Poznan, Poland; hanna.turlewicz@up.poznan.pl (H.T.-P.); agata.augustyniak@up.poznan.pl (A.A.); 2 VETPOL SP. Z.O.O., Grabowa 3, 86-300 Grudziądz, Poland

**Keywords:** hepatitis E virus, hepatitis E, zoonotic pathogen, livestock, pigs, cattle, small ruminants, rabbits

## Abstract

**Simple Summary:**

Hepatitis E virus (HEV) is a public health problem worldwide, as it is an important food pathogen that humans can obtain from animals. The most common way to infect humans is by consuming contaminated, undercooked meat or raw meat from infected pigs. However, domestic cattle, small ruminants such as sheep and goats, and farm rabbits should not be underestimated as possible sources of HEV infection for humans. Many studies have detected HEV in milk from infected ruminants. Thus, the consumption of raw milk might lead to infection. Among livestock, chickens are susceptible to avian HEV, which can cause symptomatic disease but is not dangerous to humans. Avoiding eating undercooked meat from certain livestock species and following basic hygiene rules when in contact with animals that may be a source of HEV are effective preventive measures for hepatitis E in humans.

**Abstract:**

Hepatitis E virus (HEV) is a public health problem worldwide and an important food pathogen known for its zoonotic potential. Increasing numbers of infection cases with human HEV are caused by the zoonotic transmission of genotypes 3 and 4, mainly by consuming contaminated, undercooked or raw porcine meat. Pigs are the main reservoir of HEV. However, it should be noted that other animal species, such as cattle, sheep, goats, and rabbits, may also be a source of infection for humans. Due to the detection of HEV RNA in the milk and tissues of cattle, the consumption of infected uncooked milk and meat or offal from these species also poses a potential risk of zoonotic HEV infections. Poultry infected by avian HEV may also develop symptomatic disease, although avian HEV is not considered a zoonotic pathogen. HEV infection has a worldwide distribution with different prevalence rates depending on the affected animal species, sampling region, or breeding system.

## 1. Introduction

Hepatitis E is an important public health problem worldwide. The World Health Organization estimates that 20 million infections with *Paslahepevirus balayani* (hepatitis E virus—HEV) occur worldwide, resulting in an estimated 3.3 million symptomatic cases and 44,000 deaths due to hepatitis E [1]. According to the European Centre for Disease Prevention and Control, the number of confirmed HEV cases in humans across Europe has increased yearly from 514 in 2005 to 5617 cases in 2015 [2]. Previously, HEV was thought to be limited to developing countries. Currently, it is known to be endemic as a zoonotic infectious agent in most developed and high-income countries. However, the true prevalence among humans is unknown due to frequent asymptomatic or unrecognised infections. For instance, it is estimated that in England, there are 100,000 infections with HEV per year [3], whereas in 2013, only 691 cases were laboratory-confirmed [4]. The worldwide HEV seroprevalence among humans in the available literature varies depending on the population group tested or geographical region and ranges from 0.6% to 52.5% [5,6,7,8]. HEV-1 and HEV-2 genotypes cause acute hepatitis outbreaks in humans, predominantly in developing countries, where they are spread orofaecally, mainly via contaminated water supplies [9]. In developed countries, HEV-3 and HEV-4 have been found in all stages of the human food chain, and one established route of transmission from livestock to humans is via undercooked or uncooked pig meat products [9]. Wild boars are often reservoirs of this zoonotic pathogen in wildlife, and consuming game could also be the route of HEV infection, although this route is probably less important [9]. Livestock animals other than domestic pigs may also contribute to the spread of zoonotic HEV genotypes to humans, especially cattle, goats, sheep, and farm rabbits [10,11,12,13,14,15]. HEV displays an extremely wide range of hosts, and the list of animal species proven to be susceptible to infection has expanded over the last two decades. High HEV seroprevalence in domestic pigs indicates high viral circulation among these animals, and this is associated with the increased risk of human infection through direct contact with and consumption of undercooked infected pork products [16]. People who work with livestock and have direct contact with animals (e.g., farmers, vets) have higher HEV-related seroprevalence rates than the general population [17]. All livestock professionals should be aware of the risk of zoonotic-acquired hepatitis E. Recent changes in HEV epidemiology might be related to the zoonotic nature of the disease.

In this paper, a bibliographic review of the literature on HEV is performed to gather the latest data regarding the prevalence among livestock species worldwide and the threat to humans due to HEV in livestock.

## 2. Taxonomy

The HEV genome contains a positive-sense monopartite RNA of 6.4–7.2 kb with three open reading frames (ORF1, ORF2, and ORF3) [18]. The rapidly growing number of recently proven HEV-infected animal species resulted in an inevitable revision of the previously adopted taxonomy due to numerous species remaining unassigned at the genus level in the former HEV taxonomy. HEV includes variants able to infect humans and several mammalian species. According to the 2021 release of the International Committee on the Taxonomy of Viruses, HEV belongs to the *Hepeviridae* family, which is divided into two subfamilies: *Orthohepevirinae* and *Parahepevirinae* [19]. Members of the *Orthohepevirinae* subfamily are distinct by phylogenetic analysis and have been detected in mammals and birds; members of the subfamily *Parahepevirinae* have only been detected in fish. Among the *Orthohepevirinae* subfamily, members of the *Paslahepevirus* and *Rocahepevirus* genera can infect humans and numerous domestic and wild mammals. In contrast, members of the *Avihepevirus* and *Chirohepevirus* genera can infect birds and bats, respectively. The *Paslahepevirus* genus contains two species: *P. balayani* (formerly *Orthohepevirus* A) and *Paslahepevirus alci,* which was detected in moose [18,19]. Eight different genotypes have been assigned to the species *P. balayani*: HEV-1 to HEV-8 [20]. HEV-1 to HEV-4 genotypes are commonly associated with HEV infection in humans. Of these genotypes, HEV-1 and HEV-2 are restricted to humans, whereas HEV-3 and HEV-4 are considered zoonotic. HEV-3 and HEV-4 are further classified based on phylogenetic grouping. Currently, a total of three main clades of HEV-3 can be distinguished: clade 3.1, which includes subtypes a, b, c, h, i, and j; clade 3.2, which includes subtypes e, f, and g; and clade 3.3, which contains rabbit strains corresponding to the HEV-3ra subtype [21,22,23]. Nine subtypes were determined among HEV-4: 4a–4i [20]. Subtypes 4a, 4b, 4d, and 4 h are isolated in over 80% of cases [24].

## 3. Prevalence of HEV in Livestock

### 3.1. Pigs

Domestic pigs (*Sus domesticus*) represent the most important reservoir of the zoonotic genotypes HEV-3 and HEV-4 [25]. Numerous research shows that most HEV infections in pigs are caused by HEV-3, which is widely spread across the globe (Table 1), while HEV-4 is less prevalent in the pig world population (Table 1). Nevertheless, in some countries, especially in the Western Pacific region, like China, India, or Indonesia, HEV-4 is as prevalent as HEV-3 or represents a predominant genotype. Susceptible pigs acquire HEV mainly from other shedding pigs. However, swine are also susceptible to HEV-3 and HEV-4 isolated from humans [26]. Moreover, experimental studies show that infection with HEV-3 from rabbits is also possible in these animals [27,28]. Nevertheless, no evidence of natural cross-species transmission has been provided [29]. Unusually, a human HEV-1 strain was reported in a domestic pig in 2018 in Uruguay, although pigs are not considered a natural reservoir for this genotype. Molecular methods quantified the viral load in the stool, and sequence analyses were performed. The investigated animal was simultaneously infected with HEV-3 and appeared not to have clinical symptoms. The authors suggest that pigs may occasionally, and perhaps accidentally, act as reservoirs for HEV-1 through an inter-species transmission mechanism. Detection of both genotypes in one animal means multiple sources of infection. Pigs are omnivorous; hence, a plausible alternative source of contamination could be through the consumption of contaminated food [30]. The remaining genotypes (HEV-2 and HEV-5 to HEV-8) have not been documented in pigs yet.

Zoonotic HEV-3 and HEV-4 in pigs are spread worldwide. Circulation of these genotypes has been documented in farms and slaughterhouses from countries of all continents except Antarctica. HEV-3 is widely distributed in pigs from many geographic areas, including the Americas, Europe, Africa, Japan, Southeast Asia, and Oceania, whereas HEV-4 has been reported in pigs from China, Japan, Indonesia, and some European countries. The exact data on this subject are presented in Table 1. Infections with different genotypes of HEV have little impact on animals’ health, and, in most cases, they have no apparent symptoms. Due to the subclinical course of the infection, there is no surveillance of infected herds. However, the level of enzootic HEV infections is suspected to be extremely high. Despite the lack of routine monitoring of the occurrence of HEV, breeders and consumers of meat and meat products need to be aware of the occurrence of this pathogen and its possible threat to public health. According to the review provided by Li et al. [31], nearly 60% of domestic pigs worldwide have undergone HEV infection [31]. The seroprevalence for individual countries differs, ranging from 9.90% in Thailand to 84.02% in India [31]. Besides Thailand, lower than 50% seroprevalence was observed in France, Ireland, Lithuania, Poland, Portugal, Serbia, Spain, Mexico, the USA, Argentina, Uruguay, and Cameroon [32]. Other countries where serological tests were performed displayed an overall seroprevalence of more than 50% [31]. The high seroprevalence in many countries worldwide indicates the endemic occurrence of zoonotic genotypes of HEV.

Several factors may contribute to the variation in prevalence of HEV RNA and anti-HEV antibodies across studies. Among them, the country or region of sampling has the most significant influence. The highest HEV-3 RNA prevalence was noted in Nigeria (76.6%); it was much higher in comparison with the remaining countries in which molecular studies were performed, whose prevalence was between 0.93% (Indonesia) and 49.48% (Denmark) [31]. As mentioned previously, the level of HEV seroprevalence, which may be associated with HEV circulation, depends on different farming systems, including confined or conventional, free-range, and organic farms. In the Netherlands, HEV-specific antibodies were detected in samples from conventional, free-range, and organic pig farms, indicating the possibility of introducing this pathogen to the different farming types. However, the estimated average within-herd seroprevalence was significantly higher for pigs from organic farms than for pigs from conventional farms [33], indicating a higher probability of HEV infection in organic farms due to accessibility to the contaminated environment or longer exposure through environmental contamination. A study performed in Ghana, where free-range pig rearing is widespread, also indicates that free ranging is a significant risk factor for HEV infections, unlike conventional or confined breeding systems [34]. The pig farm density has an inseparable connection with the model of pig farming and increases with the intensification of swine production. The high seroprevalence seems to be associated with a short production cycle of fattening and a high pig population in farms. Typical industrial farms are characterized by a high density of animals, shorter farming time for fattening, many herds, and close contact among pigs, which contribute to the high HEV seroprevalence in pigs [35,36]. A high density of animals that may shed the virus linked with intensive farming is probably associated with a greater accumulation of the virus in the environment. Jemeršić et al. [37] noted the positive correlation between high HEV seroprevalence and the high density of pigs and wild boars in different regions of Croatia. Given the cross-species transmission ability of HEV, it is not surprising that wild boars are the main HEV reservoirs in wildlife and play a pivotal role in HEV circulation. According to Caruso et al. [35], the same HEV subtypes are circulating within the pig and wild boar populations in the same geographic area (Piedmont and Liguria regions, north-western Italy). The horizontal transmission of HEV-3 from wild boar to domestic pigs has been proven experimentally [38]. On the other hand, swine manure might be a source of HEV infection of wild boar and other wildlife, since it is often used as a soil fertilizer. In terms of public health, the density of the pig population in a given region does not appear to be associated with a higher risk of HEV seropositivity in humans living in Germany and Denmark [39,40].

Pigs, like humans, are frequently infected with HEV via the faecal-oral route. Nucleic acids of the virus were also detected by nested RT-PCR from inside and outside farm buildings, on trucks, and in utility vehicles. Hence, the indirect transmission of the pathogen, especially during the movement of trucks and utility vehicles, plays an important role in HEV dissemination at a slaughterhouse site and throughout an entire network [41]. The infection is usually asymptomatic, and mild to moderate liver and lymph node changes can be found in infected animals [42]. Typically, the viral load peaks at 4–6 months of age, when passive immunity wanes. Infected pigs start shedding the virus about a week after exposure, for an average of 3 weeks. Viremia persists for 1–2 weeks. Shedding the virus with faeces and other excretions, e.g., urine, is of great epidemiological importance. The primary factor that plays a pivotal role in spreading and maintaining infection within the population in different farms is the accumulation of the virus in the environment [32]. HEV can probably persist in the farm environment for an extended period. HEV-3 shows high stability on different surfaces (steel, wood, plastics, and ceramics) [43]. At 23 °C, the remaining infectious virus was detected until week 4 on most surfaces and was completely inactivated after 8 weeks. However, at 3 °C, HEV was detectable for up to 8 weeks on most surfaces [43]. Faecal virus shedding in most pigs is cleared by 7–8 weeks after inoculation with HEV [44]. However, the duration of HEV-3 shedding in wild boars was reported to be up to 5 months, and the shedding duration in pigs might be similarly extended [38]. The presence of pig pathogens that can modulate the time course of natural HEV infections can impact the viral load in animals’ habitats and the prevalence range of HEV in the pig population. Co-infection with immunomodulatory viruses such as Porcine Reproductive and Respiratory Syndrome Virus (PRRSV) virus or Porcine circovirus-2 may result in the prolonged excretion of HEV in co-infected pigs [45]. Cao et al. [44] arbitrarily set 8 weeks after infection as the time point separating acute and chronic infections. Their results showed that immunocompromised pigs with chronic infection shed the virus for at least an additional 5 weeks beyond 8 weeks post-inoculation [44]. Moreover, HEV shedding is significantly increased and dramatically extended in pigs co-infected with PRRSV (48.6 versus 9.7 days for HEV only) [45]. The chronic shedding and the higher quantity of viral particles shed in the faeces of co-infected animals result in the accumulation of a high viral load in the environment, which creates higher and longer infection pressure on susceptible animals. What is important is that chronic infections may dramatically increase the risk of pig livers containing HEV at slaughter age [45].

The pigs’ age can influence the prevalence of HEV [46,47,48,49]. The prevalence of viral RNA in serum changes according to the age class of the animals and decreases over time, while the antibody prevalence increases due to continuous exposure. The infection is usually self-limiting, and specific humoral immunity protects the animals from reinfection [42]. Immunity does not last for life; in the study conducted on a farrow-to-finish herd in Spain, IgG antibodies lasted until 9 weeks of age in piglets born from strong serologically positive sows, while animals born from weak seropositive sows were positive only at 1 to 3 weeks of age [50]. The greatest prevalence of HEV RNA is observed in weaners [49]. It subsequently declines in growers and is the lowest in the group of fatteners [46,47,48]. The data concerning HEV RNA prevalence in sows is divergent; in some studies, the presence of HEV in this group was not observed [48]. In others, the prevalence of HEV RNA achieved high values: 38.6% (22/57) in young sows and 53.4% (31/38) in older sows that have delivered more than two litters [46]. HEV RNA is usually absent in suckling piglets, as infection occurs at later stages of life due to the protective impact of maternal immunity [46,48,49]. Nevertheless, sometimes it can be detected even in pigs aged 1–4 weeks [51]. 

**Table 1:** Detection of anti-HEV antibodies and/or RNA in porcine samples from various countries.

Country	Sample Type	Positive Samples/Collected Samples	Prevalence (%)	Detected Material	Year of Collection	Genotype	References
Africa							
Cameroon	Serum	70/162	43.21	Antibodies	2012	3	[52,53,54]
	Faeces	8/136	5.88	RNA	2017–2018		
	Liver	3/345	0.87		2012		
Democratic Republic of Congo	Faeces	1/40	2.5	RNA	2010	3	[55]
Ghana	Serum	296/474	62.44	Antibodies	2022	3	[34,56]
		26/474	5.49	Antigen			
		9/89	10.11	RNA	2011		
Madagascar	Serum	178/250	71.20	Antibodies	2010–2011	3	[57]
	Liver	3/250	1.20	RNA			
Nigeria	Serum	159/286	55.59	Antibodies	2011–2012	3	[58]
	Faeces	69/90	76.67	RNA			
Republic of South Africa	Faeces	7/160	4.38	RNA	2016	3	[59]
Zambia	Serum	231/484	47.73	Antibodies	2017–2019	3	[60]
	Faeces	8/25	32.00	RNA			
	Liver	12/100	12.00				
Asia							
Bangladesh	Serum	82/100	82.00	Antibodies	2011	ND	[61]
	Faeces	0/100	0.00	RNA			
China	Serum	528/788	67.00	Antibodies	2004–2006	3, 4	[62,63,64]
	Serum	18/394	4.57	RNA	2004–2006		
	Kidney	4/129	3.10		2017–2018		
	Liver	7/114	6.14		2017–2018		
	Blood curd	2/170	1.18		2017–2018		
	Bile	6/53	11.32		2018		
	Semen	1/26	3.85		2018		
	Faeces	26/445	5.84		2018		
India	Serum	104/160	65.00	Antibodies	2016	4	[65]
	Faeces	14/160	8.75	RNA			
Indonesia	Serum	58/101	57.43	Antibodies	2004	4	[66]
	Serum	5/101	4.95	RNA			
Japan	Serum	126/169	74.56	Antibodies	2004	3	[67,68]
		3/169	1.78	RNA			
	Bile	20/200	10.00	RNA	2020		
Korea	Serum	39/264	14.77	Antibodies	2003	3, 4	[69,70,71]
	Serum	3/128	2.34	RNA	2003		
	Faeces	30/148	20.27		2017		
	Liver	42/388	10.82		1995–2004		
Laos	Serum	152/722	21.05	Antibodies	2008–2009	4	[72,73]
	Faeces	21/181	11.60	RNA	2009		
Mongolia	Serum	223/243	91.77	Antibodies	2006	3, 4	[74,75]
	Serum	89/243	36.63	RNA			
	Faeces	30/200	15.00		2020–2022		
	Liver	4/60	6.67				
Nepal	Serum	18/55	32.73	Antibodies	1995	ND	[76]
	Serum, faeces	3/47	6.38	RNA			
Philippines	Serum	150/299	50.17	Antibodies	2010–2011	3	[77]
	Faeces	22/299	7.36	RNA			
Singapore	Serum	143/409	34.96	Antibodies	2000–2019	3	[78]
	Faeces	34/409	8.31	RNA			
	Liver	4/60	6.67				
Thailand	Serum	87/879	9.90	Antibodies	2009	3	[79,80]
	Faeces	25/875	2.86	RNA			
	Liver	1/51	1.96		2013–2014		
Vietnam	Serum	278/475	58.53	Antibodies	2017	3, 4	[81]
	Faeces	17/250	6.80	RNA	2018–2019		
Australia and Oceania							
New Zealand	Serum	54/72	75.00	Antibodies	2001	ND	[82]
	Faeces	17/45	37.38	RNA			
Europe							
Austria	Liver	5/43	11.63	RNA	2007–2009	3	[83]
	Bile	3/28	10.71				
	Kidney	1/1	100.00				
	Serum	2/42	4.76				
Belgium	Serum	306/420	72.86	Antibodies	2010–2011	3, 4	[84,85]
	Serum	4/420	0.95	RNA			
	Faeces	8/115	6.96		2008		
Bulgaria	Serum	81/225	36.00	Antibodies	2021	3	[86,87]
	Serum	11/39	28.21	RNA	2018–2020		
Croatia	Serum	55/60	91.67	Antibodies	2012	3	[88]
	Serum	8/60	13.33	RNA			
	Bile	3/37	8.11				
Czech Republic	Serum	1/31	3.23	RNA	2008	3	[89]
	Bile	12/30	40.00				
	Liver	5/31	16.13				
Denmark	Serum	156/213	73.24	Antibodies	2007–2008	3, 4	[90,91]
		167/247	67.61		2019		
	Faeces	48/97	49.48	RNA	2009		
	Caecum content	64/250	25.60		2019		
	Serum	40/248	16.13				
	Liver	25/228	10.96				
Estonia	Serum	234/380	61.58	Antibodies	2015	3	[92]
	Faeces	103/449	22.94	RNA			
Finland	Faeces	15/67	22.39	RNA	2009	3	[93]
France	Serum	87/215	40.47	Antibodies	2007	3	[94,95]
	Serum	22/215	10.23	RNA	2007		
	Faeces	65/207	31.40				
	Rectal swabs	34/50	68.00		2012		
Germany	Serum	1065/2273	48.85	Antibodies	2011	3	[96,97]
	Liver	8/200	4.00	RNA	2011		
Greece	Serum	76/96	79.17	Antibodies	2002	ND	[98]
Hungary	Faeces	30/132	22.73	RNA	2005–2006	3	[99]
	Liver	12/39	30.77				
Ireland	Serum	89/330	26.97	Antibodies	2010–2011	ND	[100]
Italy	Serum, meat juice	314/409	76.77	Antibodies	2017–2019	3	[101]
	Liver	12/585	2.05	RNA	2017–2019		
	Diaphragm	8/21	38.10		2017–2019		
	Faeces	11/569	1.93		2017–2019		
Lithuania	Serum	168/384	43.75	Antibodies	2014–2015	ND	[102]
Netherlands	Serum	687/846	81.21	Antibodies	2004	3	[33,84]
	Faeces	16/101	15.84	RNA	2008		
Norway	Serum	484/663	73.00	Antibodies	1994, 2009, 2020	ND	[103]
Poland	Blood	5/146	3.42	RNA	2018–2019	3	[104]
	Liver	1/100	1.00		2016–2017		
Portugal	Faeces	44/200	22.00	RNA	2010–2011	3	[105]
Romania	Faeces	6/19	31.58	RNA	2009–2010	3	[106]
Russian Federation	Faeces	34/219	15.53	RNA	2007–2016	3	[49]
Serbia	Serum	162/339	47.79	Antibodies	2016–2018	3	[107,108]
	Liver	51/330	15.45	RNA	2018		
Slovakia	Rectal swabs	53/388	13.66	RNA	2017	3	[48]
Slovenia	Faeces	15/85	17.65	RNA	2004–2005	3	[109,110]
	Faeces	44/811	5.43		2014		
	Bile	43/811	5.30				
	Liver	40/811	4.93				
Spain	Serum	1390/2871	48.42	Antibodies	1985–1997	3	[111,112]
	Serum	3/45	6.67	RNA	2017		
	Faeces	6/45	13.33				
	Liver	7/45	15.56				
	Kidney	5/45	11.11				
	Heart	4/45	8.89				
	Diaphragm	1/45	2.22				
Sweden	Faeces	71/240	29.58	RNA	2010	3	[113]
Switzerland	Serum	1161/1999	58.08	Antibodies	2006, 2011	3	[114,115]
	Liver juice, diaphragm juice	15/54	27.77		2018		
	Liver	1/192	0.52	RNA	2018		
	Liver	7/54	12.96				
	Diaphragm	4/7	57.14				
	Faeces	7/7	100.00				
United Kingdom	Serum	108/176	61.36	Antibodies	2006	3	[116,117]
	Serum	72/162	44.44	RNA	2006		
	Faeces	42/195	21.54		2007		
North America							
Canada	Liver	2/19	10.53	RNA	2014–2015	3	[118]
Cuba	Serum	165/187	88.24	Antibodies	2016	3	[119,120]
	Faeces	16/173	9.25	RNA	2016		
	Faeces	10/53	18.87		2007		
Mexico	Serum	100/125	80.00	Antibodies	2004	3	[121]
	Serum	8/125	6.4	RNA			
	Faeces	28/92	30.43				
United States	Serum	2007/5033	39.88	Antibodies	2017–2019	3	[122]
	Serum	318/5033	6.32	RNA			
South America							
Argentina	Faeces	11/135	8.15	RNA	2018	3	[123]
Bolivia	Faeces	7/22	31.82	RNA	2006	3	[124]
Brazil	Liver	2/118	1.69	RNA	2010	3	[125,126]
	Bile	1/118	0.85				
	Faeces	2/119	1.68		2017		
Colombia	Serum	1000/1000	100.00	Antibodies	2011–2012	3	[127,128]
	Liver	109/300	36.33	RNA	2011–2012		
	Faeces	110/300	36.67				
Uruguay	Serum	103/220	46.82	Antibodies	2012–2016	3	[30]
	Liver	25/150	16.67	RNA	2017		

### 3.2. Cattle

**Figure 1:**
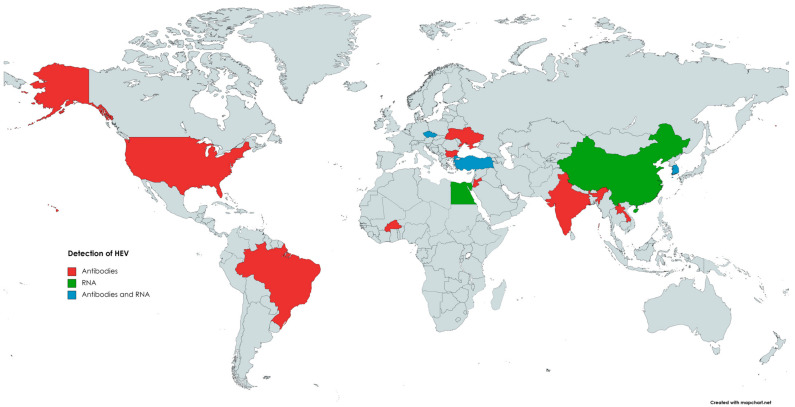
The occurrence of anti-HEV antibodies and/or HEV RNA in cattle.

The first evidence of HEV circulation in the cattle population was reported in 1998 when anti-HEV antibodies were found in bovine samples from Somalia, Tajikistan, Turkmenistan, and Ukraine [129]. Since then, the presence of anti-HEV antibodies in cattle has been reported in the Americas, Africa, Asia, and Europe (Figure 1). Evidence of HEV infection and/or contact with this pathogen was found in cattle (*Bos taurus*), as well as in yak (*Bos grunnicus*) and buffaloes (*Bison bison*, *Syncercus caffer*) [130,131,132,133]. The genotypes of HEV detected in cattle were mainly 3 and 4, although HEV-1 was also found in bovine milk in a study performed in Türkiye [10]. HEV could be transmitted from farm ruminants via contact with farmworkers or their contaminated workwear [134]. Contamination of drinking water with animal manure is considered the primary source of HEV infection in humans and animals in developing countries [135]. In the study by Tritz et al. [136], HEV infections were more prevalent in animals with open access to river water.

In China, between 2002 and 2018, the seroprevalence of HEV-specific antibodies ranged from 6.3% (12/190) [137] to 47% (120/254) [62,138,139,140]. Anti-HEV antibodies were also detected in cattle milk, with a prevalence of 14.9% (40/269) [141]. HEV RNA was also found in other studies conducted in China, with the prevalence ranging from 0% (0/200) [142] to 3% (8/254) in serum [134], from 8.8% (8/91) [143] to 37.1% (52/140) [12] in faeces, and from 0% (0/269) [135] to 100% (52/52) [12] in cattle milk. The seroprevalence in India reached 4% (4/91) and 6.9% (13/188) [144], 6.8% (11/161) in Lao People’s Democratic Republic [136], and 14.5% (18/124) in Jordan [145]. All the faecal swabs from cattle from rural settings in Lao People’s Democratic Republic that were subjected to PCR analyses were negative (0/173) [136]. In Korea, HEV RNA was found in 1% (1/100) of the examined bovine liver samples [11].

In Europe, anti-HEV antibodies were found in cattle for the first time in 1998 in Ukraine [129]. A recent study conducted in Bulgaria showed that the seroprevalence in these animals reached 7.7% (14/180) [146]. Neither anti-HEV antibodies in bovine sera nor HEV RNA in bovine faeces were found in Spain [147]. The bovine faeces samples in Hungary (0/125) [51], blood and liver bovine samples in Croatia (0/30) [148], and milk samples in Germany (0/400) [149] and Belgium (0/275) [150] were also free from HEV RNA. On the other hand, 29.2% (14/48) of bovine milk samples were positive for HEV RNA in the study conducted in Türkiye [10].

In the United States, where 983 bovine serum samples were investigated, the seroprevalence rate across herds from different regions ranged from 16.1% to 68.4%, with an overall rate of 20.4% [151]. In Brazil, the seroprevalence reached 1.4% (1/70) [152]. 

In Africa, the seroprevalence reached 21.6% (11/51) in Egypt [132] and ranged from 5.1% (24/475) [153] to 26.4% (19/72) in Burkina Faso [154]. Anti-HEV antibodies were not found in the bovine sera sampled and examined in Nigeria (0/37) [155].

Variations in the prevalence of HEV in cattle observed by different researchers may result from different breeding practices, of which the most critical factor influencing the increase in the prevalence of HEV is mixed breeding, including for many species. For domestic cattle, sheep, and goats, the incidence of HEV infections appears to be higher in rural areas where traditional mixed farming systems consist of small family farms with pigs and other domestic animals [51,133,137,156]. Cross-species transmission between ruminants and pigs is suggested by the detection of HEV-3 and HEV-4 that are genetically closely related to the HEV identified in domestic and wild ruminants and pigs or wild boars in the same geographical areas [133,140,156]. Moreover, the frequent mixed breeding of cattle and pigs in rural China and the simultaneous lower seroprevalence rates in cattle compared with pigs in the same region are also probably related to cross-species transmission of the virus [140]. However, keeping cattle and pigs in close contact did not always contribute to infection; HEV RNA was not detected in the faeces and milk of cows reared on mixed farms, rearing pigs and cattle, in China [63] and Belgium [150]. In a longitudinal study, the lower seroprevalence in cattle than in pigs may be related to the lower permissivity and/or transient seropositivity observed in calves and adult cows [151]. The lack of association between the age of the cattle and seropositivity, observed in the study of Tialla et al. [153], can be explained by temporary seropositivity. According to Tialla et al. [153], the type and intensity of interspecies contacts and the hygiene prevailing on the farm may also influence the prevalence of anti-HEV antibodies in cattle [153]. Farm rabbits are also considered a reservoir of HEV; however, keeping rabbits and cattle in close contact on the same farm has not been identified as a risk factor for an increased HEV seroprevalence [153]. HEV RNA was also found in sheep kept close to investigated yellow cattle. Genome sequences of HEV strains isolated from the yellow cattle exhibited 95.1–99.8% homology with sheep-derived strains. This suggests the existence of complex mechanisms for the interspecies transmission of HEV and its circulation between populations of different animal and human species [140].

### 3.3. Small Ruminants

Sheep (*Ovis aries*) are susceptible to HEV infection, as biochemical and histological symptoms of hepatitis, shedding of the virus in faeces, and the presence of HEV RNA in the parenchymal organs in the inoculated lambs were observed [157]. Anti-HEV antibodies and HEV RNA in various ovine samples (serum, liver, faeces, milk) confirmed this finding [15,140,158].

**Figure 2:**
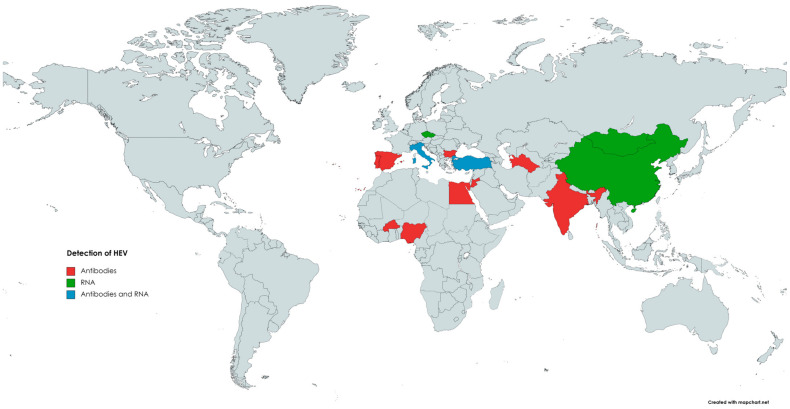
The occurrence of anti-HEV antibodies and/or HEV RNA in sheep.

The occurrence of HEV in small ruminants has been previously reviewed by Di Profio et al. [159]. According to the authors, seropositive sheep were found in numerous countries on different continents (Figure 2), such as Asia (India, China, Jordan), Europe (Spain, Italy, Portugal, Bulgaria), and Africa (Egypt, Nigeria, Burkina Faso) [146,159]. The highest seroprevalence was observed in India, with 100% or 77.5% positivity, depending on the assay used. However, an inhibition assay performed in this study did not confirm the specificity of the detected antibodies. Hence, the seroprevalence in Indian sheep can be overestimated [130]. The lowest seroprevalence was noted in Spain, as two separate reports indicated seropositivity at the level of 1.9% and 2.1%, respectively [130,147,160]. In the remaining countries, seropositivity among sheep ranged between 4.4% and 42% [159]. Results obtained in Brazil demonstrated that none of the ovine serum samples were positive for anti-HEV antibodies [158].

The first detection of HEV RNA in sheep occurred in 2010 in China; viral genetic material was detected in 2 out of 115 (1.7%) serum samples [139]. Subsequent studies confirmed that HEV was circulating in the Chinese sheep population, as HEV RNA was present in the serum (11.4%) as well as liver (5.3%) samples [15,140]. Phylogenetic analysis revealed that strains obtained from sheep livers belonged to genotype 4, subtype 4d, and shared the highest identity with human and swine HEV strains [15]. HEV genetic material was also detected in ovine specimens in several other countries (Figure 2). Available data indicate that HEV is highly prevalent in the population of Italian small ruminants [161]. Two different reports demonstrated the presence of HEV RNA in the serum and faecal samples collected from sheep [158,161]. The first study obtained serum and faecal specimens from 192 sheep [151]. HEV RNA was detected in 10.4% (20/192) of the faecal samples; in addition, 3 out of these 20 positive sheep were also viremic. Isolated strains were classified into genotype 3, subtype c, and showed a high identity level to those identified in goats, wild boars, and humans [158]. In the subsequent study, faecal samples were collected from clinically healthy sheep derived from farms located in different regions of Italy to farms from previous studies (north versus south of the country). HEV RNA was found in specimens from two seropositive farms, and the overall prevalence reached 3% (4/134) [161]. The most recent data come from Mongolia, in which HEV genetic material was detected in 5% (3/60) of sheep livers and 2% (4/200) of sheep faeces [75]. Sequencing of genetic material revealed that Mongolian isolates belonged to genotype 4 and were highly homologous to those detected in pigs [75].

**Figure 3:**
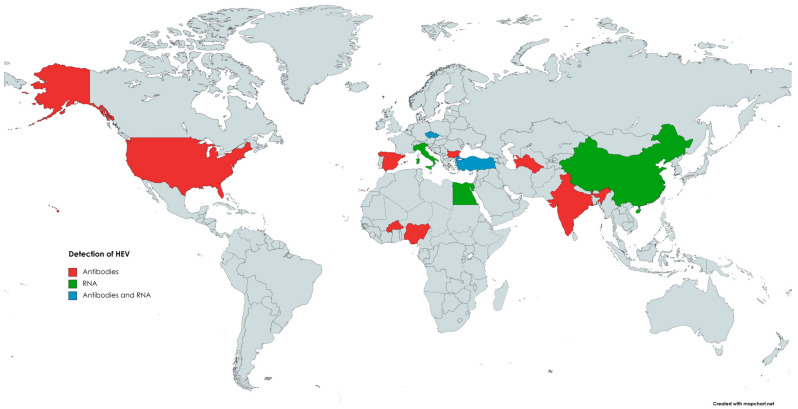
The occurrence of anti-HEV antibodies and/or HEV RNA in goats.

The first evidence indicating the susceptibility of goats (*Capra hircus*) to HEV infection comes from 1998, when anti-HEV antibodies were detected in caprine samples [129]. In 2007, in India [130], 100% (98/98) of tested caprine sera were positive for anti-HEV antibodies [130]. Since then, many reports from numerous countries (Figure 3), such as China, the USA, Egypt, Nigeria, Italy, Spain, and Bulgaria, confirmed the presence of antibodies against HEV in the sera of goats, with seroprevalence ranging from 0.6% to 46.7% [146,159].

The first HEV RNA detection in caprine specimens has been reported by Di Martino [156]. Noteworthily, previous studies conducted in China in which samples collected from seropositive goats were also tested for the presence of HEV RNA failed to detect HEV genetic material; nevertheless, in one of them, by using a monoclonal antibody-based enzyme immunoassay, HEV antigens were found in caprine sera [62,142,162]. In the Italian study, 9.2% (11/119) of faecal samples collected from small goat farms were positive for viral RNA [149]. The obtained strains have been classified into animal and human HEV-3, subtype c, with the closest nucleotide identity (94.2–99.8%) being to the wild boar strain identified in the same geographical area [156]. The high HEV prevalence in goats was also observed in the Yunan Province of China, where raw mutton and goat milk are traditionally consumed [163]. Results of this study showed that 74.04% (40/54) and 60% (12/20) of faecal samples were HEV-RNA-positive. These values were significantly higher than those in cattle (37.14%) in the same area. Moreover, 53.57% (15/28) of tested goats were viremic. Abundant amounts of HEV antigens were also found in the livers and spleens of infected goats. A slight increase in alanine aminotransferase and aspartate aminotransferase and histopathological liver damage in infected animals were also observed. Strains obtained from goats have been classified as HEV-4, subtype 4, and shared high similarity (>99.6%) with human, swine, and bovine HEV strains in the same area [163]. Another study demonstrated the presence of HEV RNA in 4% (2/50) of caprine liver samples collected at slaughterhouses in the Tai’an region, China [13]. Obtained isolates were classified into HEV-4, subgenotype 4f, and were closely related (91.2–93% nucleotide sequence identity) to cow HEV stains from the same province [13]. In Egypt, HEV RNA was detected in two out of five fresh liver samples collected from HEV seropositive goats [164]. In addition, similarly to cattle, small ruminants can spread HEV through milk. The presence of HEV RNA in caprine milk was noted in several countries, such as Türkiye and Egypt, with a prevalence of 18.46% (12/65) and 0.71% (2/280), respectively [10,164]. HEV RNA was also detected in 4 out of 240 pooled samples of caprine milk, including 2674 goat milk samples from 128 goat farms in the Czech Republic. In the same study, HEV RNA was also found in 4 out of 50 pooled samples of ovine milk from 938 sheep from 12 different farms [165]. Isolates from Egypt were classified into HEV-3, subtype 3a, and one of these isolates displayed high homology to the HEV obtained from cow milk in the same geographic area [164]. The high levels of nucleotide sequence identities among HEV isolated from samples collected from goats and other animal species in the same area may indicate cross-species virus transmission [13,163]. Considering the above, keeping goats and sheep together with other species susceptible to HEV infection, such as cattle or pigs, may be a risk factor for HEV infection. Small ruminants can also acquire an infection with human HEV, possibly after contact with contaminated water or feed. The difference in HEV prevalence between sheep and goats may result from varying susceptibility between species or behavioural or ethological differences. HEV is excreted in faeces [156,158], and, unlike sheep, goats frequently jump inside or insert their feet into feeders and drinkers, which may increase the risk of transmission of HEV via the faecal–oral route [160].

### 3.4. Rabbits

Next to pigs, rabbits (*Oryctolagus cuniculus*) are considered another major HEV reservoir [166]. These animals are a natural host of the rabbit HEV strain (HEV3-ra), currently classified within the HEV-3 genotype [167,168,169]. Based on phylogenetic analysis of the complete genome, some researchers considered HEV3-ra to be a separate genotype due to its low nucleotide sequence identity [167,168]. Besides HEV3-ra, rabbits are also vulnerable to infections with different HEV genotypes, such as human-derived HEV-4, swine-derived HEV-4, and camel-derived HEV-8. However, this susceptibility was assessed only during experimental studies, and, currently, there is a need to investigate further the natural infection with the mentioned genotypes in rabbits [169,170]. These can become infected with HEV via the faecal–oral route. Nevertheless, vertical transmission is also possible [171]. During natural infection, elevated levels of ALT and pathological changes in the liver have been observed [171]. Transient viremia and seroconversion can also occur [171]. High viral loads are shed with faeces for up to 22 weeks [171]. Under experimental conditions, faecal shedding can even last for nine months [172]. HEV3-ra has zoonotic potential, and cross-species transmission to pigs, mice, and *cynomolgus macaques* has been documented for this virus [28,173,174].

**Figure 4:**
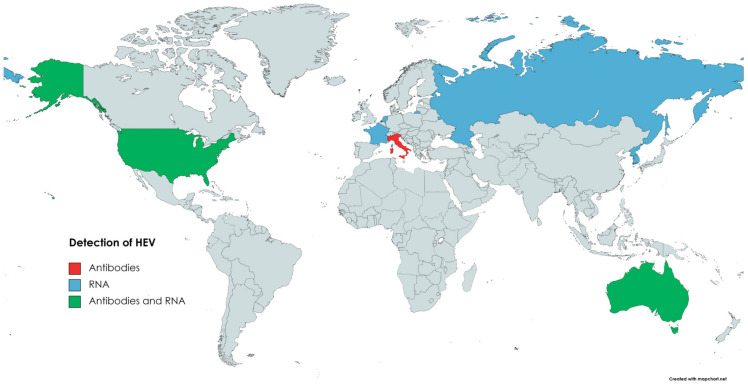
Circulation of HEV in domestic rabbits.

HEV from rabbits was isolated for the first time in 2009 in China [167]. Viral RNA was detected in serum samples of rex rabbits from two farms, and 7.5% (25/335) of the samples were positive for HEV RNA. Moreover, anti-HEV antibodies were found in 57% (191/335) of the sera [167]. Subsequent studies confirmed that HEV3-ra circulates in the Chinese rabbit population [168,175]. The presence of HEV in rabbits was also confirmed in several other countries (Figure 4). In Korea, HEV RNA was detected in specimens collected from farm rabbits. Positive faecal samples came from two out of six examined rabbit farms; the prevalence was 6.4% (17/264) [176]. Several of the reports on HEV infection in rabbits come from Europe. Initially, a retrospective study performed in France showed that 7% (14/200) of bile samples from farm rabbits and 23% (47/205) of liver samples from wild rabbits were positive for HEV RNA [177]. In the Netherlands, HEV prevalence in the faeces of pet rabbits was 23% (8/35) [178]. Interestingly, none of the samples collected from farm rabbits were positive in this study. However, this could result from the low number of samples tested (*n* = 10) [178]. The seroprevalence of HEV in Italian farm and pet rabbits was assessed as 3.4% and 6.56%, respectively [179]. Despite anti-HEV antibodies, none of the samples (serum, faeces) were HEV-RNA-positive. Nevertheless, the above data indicate that HEV circulates in the Italian rabbit population [179]. The presence of HEV RNA was also confirmed in the samples collected from farm rabbits in Russia [49]. Three out of six surveyed farms were HEV-positive, and the virus was detected in 9 out of 206 faecal samples [49]. In contrast, despite testing 372 liver samples, no HEV circulation in wild rabbits has been found in Spain [180]. HEV genetic material and anti-HEV antibodies were confirmed in faecal and serum samples collected from two rabbit farms in Virginia, USA [181]. A total of 16.5% (14/85) of serum samples and 15% (13/85) of faecal samples were HEV-RNA-positive. Antibodies against HEV were found in 36% (31/85) of the sera samples [181]. In Australia, HEV circulation has been described in the wild and domestic rabbit population, as HEV RNA and anti-HEV antibodies have been detected in collected samples (sera, livers) [181,182].

### 3.5. Poultry

Members of the genus *Avihepevirus* are phylogenetically distinct from other viruses in the *Orthohepevirinae* subfamily and have a different host range. It has been found only in birds. *Avihepevirus magniiecur* (avian HEV, previously known as *Orthohepevirus B*) includes strains of avian HEV detected in chickens [183,184,185,186]. In chickens, infection with avian HEV has been associated with big liver and spleen disease, hepatic rupture, haemorrhage syndrome [187], diarrhoea, ovarian regression, and other disorders [184]. However, hepatitis E in poultry is often subclinical, making it difficult to detect in the flock and masking the circulation of the virus between susceptible birds. Avian HEV has been successfully reported worldwide: in the United States [185], Canada [185], Brazil [152], China [188], Korea [189], Taiwan [190], Russia [191], Poland [192], Hungary [193], Austria, the Czech Republic [194], and Spain [195]. It has also been detected in Australia [196] and Africa [197].

The available evidence suggests that avian HEVs are likely not zoonotic. The phylogenetic analysis does not indicate a close relationship with strains pathogenic to humans [42]. Moreover, experimental inoculation of rhesus monkeys with avian HEV did not result in infection. Therefore, it was assumed that avian HEV is not likely to infect primates—they showed no viremia or antibody production after inoculation [198]. The primary hosts of avian HEV are broiler breeders and layers of the *Gallus gallus domesticus*; however, these viruses have also been identified in other poultry species [188]. Under experimental conditions, turkeys (*Meleagris gallopavo*) were successfully infected with avian HEV, as evidenced by their seroconversion to anti-HEV antibodies, viremia, and faecal virus shedding [199]. Various animal species kept close to infected poultry may seroconvert, indicating exposure to the virus and the development of a humoral response. In a study conducted in China, 8/16 rabbits, 9/30 ducks, and 6/24 geese in the investigation were positive for anti-avian HEV antibodies. Part of the faecal swabs in rabbits, ducks, and geese were positive in a PCR targeting the detection of avian HEV ORF1 or ORF2 sequences [188]. Avian HEV has only 50% similarity to HEV sequences isolated from humans or pigs; nevertheless, HEVs found in birds and pigs are ubiquitous viruses. There is likely frequent contact between each other, creating the potential for mutations that may contribute to crossing the species barrier and enabling the replication of avian HEV in pigs.

## 4. Livestock as a Source of Zoonotic HEV

The range of animals proven to be susceptible to HEV infection has grown over the past two decades. The susceptibility of animals to infection is determined based on the detection of viral RNA in the samples taken from these animals and the possibility of developing a humoral response against the pathogen in response to infection detected with serological tests [42]. Hepatitis E in humans and its etiological agent were described in detail in 1983 [42]. To date, it has been detected in the human population in almost every country [200]. Later studies showed over 90% similarity between human HEV strains and those obtained from pigs [76,201]. Humans are vulnerable to infection from HEV-1 to HEV-4, representing the main reservoir of HEV-1 and HEV-2. Zoonotic transmission is widely documented for HEV-3 and HEV-4, and one case of human infection with HEV-7 with a zoonotic origin has been described [202].

HEV zoonotic infection may be foodborne or due to direct contact with infected animals [203,204]. Pigs are considered the main and apparent reservoirs of HEV-3 and HEV-4. These genotypes have been found in all stages of the human food chain, and the main route of transmission from pigs to humans is via undercooked or uncooked porcine meat products and offal [9]. It was reported that approximately 2% and 11% of pig livers sold in Japan and America, respectively, are positive for HEV RNA [205,206]. Besides foodborne transmission, direct contact with porcine body excretions may be another route for zoonotic HEV, since it has been detected in nasal and rectal swabs [207] and urine specimens [208] from experimentally infected pigs. However, animal species other than pigs may significantly contribute to the spread of zoonotic HEV genotypes to humans. Cattle and small ruminants are the source of food for humans. The possibility of cattle-to-human HEV transmission by drinking contaminated unpasteurised milk [12] or eating contaminated undercooked meat [140] and offal (e.g., liver) [11] raises the need for surveillance in this host. Go et al. [11] detected the RNA of HEV-4 in 1% of bovine liver samples (1/100) purchased from local grocery markets between February 2017 and July 2018 in Seoul, Korea. The nucleotide sequence indicated that the found HEV genotype was closely related (95.4–99.6% nucleotide identity) to human HEV-4 strains reported from Korea (FJ763142) and China (KC492825) [11]. Recently, HEV was also detected in bovine liver samples from slaughter in Brazil, where 5.41% positive samples were observed (13/240). One of the samples was identified as HEV-3 [209]. Huang et al. [12] investigated fresh pasteurised milk available at the market supplied by local farmers in China. A gavage of raw or pasteurised milk contaminated with HEV led to active infection in rhesus macaques and the detection of HEV RNA in their faeces and blood. Interestingly, pasteurisation could not inactivate HEV, and the gavage of pasteurised milk resulted in an active HEV infection in monkeys. However, Huang et al. [12] proved that short-time boiling at 100 °C for 3 min could inactivate the HEV completely. Viral RNA was not detectable in either faeces or serum of the rhesus macaques inoculated with cooked samples containing HEV material [12]. Homology analysis based on the complete sequence of the bovine HEV discovered in this study indicated that it shared 99.2~99.4% similarity to human HEV. Phylogenetic analysis revealed that all the HEV isolates from cow/milk belong to genotype 4 and subtype 4 h [12]. Similarly to cattle and goats, sheep can spread HEV through mammary gland secretions. Two separate studies from Türkiye and the Czech Republic indicated the presence of HEV genetic material in sheep’s milk; 12.3% and 1.4% of analysed specimens were positive, respectively [10,165]. The above findings are of great importance and indicate that milk from farm ruminants, especially when raw, represents a potential source of infection for consumers [165]. Data from China demonstrated a significantly higher level of anti-HEV antibodies among rabbit slaughterhouse workers than in the general population, implying a risk of cross-species transmission of HEV from rabbits to humans by direct contact [210]. The HEV-3ra strain [211] was detected in immunocompromised and immunosuppressed human patients [212,213]. Moreover, it was reported that healthy blood donors tested positive for HEV, and an isolated virus was related to HEV3-ra [214]. Interestingly, the positive individuals had had no contact with rabbits, and only two ate rabbit products that were always well-cooked; this phenomenon can suggest waterborne transmission of HEV3-ra to humans [212,213,214]. As mentioned, human infection with the HEV-7 genotype has also been described [202]. To date, the only confirmed case of human infection with zoonotic HEV-7 was documented in a patient who regularly consumed camel milk and meat [202]. 

## 5. Conclusions

There is a strong interdependence between the health of humans and animals and the environment, which is known as the One Health concept [215]. This collaborative, multidisciplinary approach aims to promote, improve, and protect the health of all species [215]. Since infections, mainly with HEV-3 and HEV-4, can be transmitted zoonotically, they should be included in the multidisciplinary One Health concept to improve the surveillance and control of this emerging infection and to prevent the spread from animal hosts to people. Available data indicate that HEV is circulating globally in human and livestock populations, and infections with this agent in livestock animals are much more common than previously thought. Human hepatitis E associated with HEV-3 and HEV-4 of animal origin result from close contact with infected livestock or from ingesting contaminated meat products or milk and is often underdiagnosed. Therefore, the consumption of under-cooked raw meat products should be avoided, especially by individuals at high risk of developing severe hepatitis E. Pigs are the main animal reservoir of zoonotic HEVs. Infection in this species has been noted in numerous countries on almost every continent. Nevertheless, viral RNA of HEV-3 and HEV-4 and/or anti-HEV antibodies have also been detected in samples collected from other species of farm animals, including cattle, sheep, goats, and rabbits. Due to the possibility of cross-infection, domestic pigs, ruminants, rabbits, and even poultry should be kept without close contact. Avian HEV poses no threat to humans, although its circulation between livestock populations may lead to changes in the genome and lead to potential infectivity to mammals. It is crucial to highlight that good hygiene practices (e.g., changing clothes or showers after dealing with farm animals) among farmworkers and other people who have close contact with livestock are advisable to minimise the risk of zoonotic HEV infection and of spreading the pathogen among susceptible animals. Knowledge concerning the epidemiology of this virus is essential in view of public health concerns. 

## Data Availability

Not applicable.
